# A review shows that ATG10 has been identified as a potential prognostic marker and therapeutic target for cancer patients based on real-world studies

**DOI:** 10.3389/fonc.2025.1573378

**Published:** 2025-04-17

**Authors:** Ke Shi, Di Ke, Feng Li, Rong-Shu Shi, Tao Liu, Dan Li, Qun-Xian Zhang

**Affiliations:** ^1^ Department of Thoracic Surgery, Beilun District People’s Hospital of Ningbo, Ningbo, China; ^2^ Department of Radiology, Affiliated Hospital of Zunyi Medical University, Zunyi, China; ^3^ Department of Cardiothoracic Surgery, Taihe Hospital, Hubei University of Medicine, Shiyan, China; ^4^ Department of Oncology, Taihe Hospital, Hubei University of Medicine, Shiyan, China

**Keywords:** ATG10, epithelial-mesenchymal transition, cancer, review, therapeutic target

## Abstract

Autophagy-related genes (ATGs) play a crucial role in tumorigenesis and cancer progression. ATG10, a member of the ATG family, has been implicated in various malignancies, including endometrial cancer, hepatocellular carcinoma, acute leukemia, nasopharyngeal carcinoma, gastric cancer and colorectal cancer. Its overexpression is frequently associated with poor prognosis and increased disease progression. ATG10 promotes cancer growth and metastasis by modulating epithelial-mesenchymal transition and cell cycle regulators such as cyclin B1, CDK1 and CDK2. However, its activity can be inhibited by several factors, including DDX10, PTBP1, sodium orthovanadate, podofilox, SIRT6, FAT1, SOX2 and multiple microRNAs (e.g., miR-369-3p, miR-100-3p, miR-27b-3p, miR-197-3p, let-7i-5p and miR-552). This review explores the functional and clinical significance of ATG10 across various cancers, highlighting its potential as a biomarker and therapeutic target.

## Background

Cancer progression involves inevitable alterations in the expression of key regulatory factors (1–5). Huang et al. reported that chondroitin sulfate synthase 3 (CHSY3) is highly expressed in gastric cancer tissues, where its overexpression correlates with poor prognosis and advanced T staging. *In vitro* and *in vivo* studies confirm that CHSY3 enhances gastric cancer cell proliferation, migration and invasion (3). Similarly, Xiao et al. identified aberrant expression of T cell receptor-associated transmembrane adaptor 1 (TRAT1) in lung adenocarcinoma, where specific mutations are associated with poor prognosis. TRAT1 overexpression suppresses cell viability, migration and invasion while promoting apoptosis. Moreover, it is significantly linked to immune cell infiltration, including B cells, CD8+ T cells and cytotoxic cells, as well as RNA modification processes (4). These findings underscore the critical roles of key genes in cancer progression.

Autophagy-related genes (ATGs) have also been implicated in tumor development and progression (6–10). In non-small cell lung cancer (NSCLC), ATG5 and circ-FOXM1 are significantly upregulated. Suppressing circ-FOXM1 inhibits NSCLC cell viability, migration and autophagy while inducing apoptosis, an effect mediated by circ-FOXM1’s regulation of miR-149-5p to enhance ATG5 expression (9). Among ATGs, ATG10 has garnered increasing research interest due to its abnormal expression in multiple cancers. Elevated ATG10 levels are strongly associated with tumor progression and poor prognosis (11–25). It is overexpressed in endometrial cancer, hepatocellular carcinoma and acute leukemia, where its upregulation correlates with reduced survival and increased disease progression. This review summarizes the molecular mechanisms and clinical significance of ATG10 across cancers, drawing on findings from clinical tissue samples, *in vitro* experiments and *in vivo* models. Our analysis highlights ATG10 as a potential biomarker and therapeutic target in oncology.

## ATG10 is overexpressed in various cancers

ATG10 expression is significantly elevated in endometrial cancer, hepatocellular carcinoma, acute leukemia, nasopharyngeal carcinoma, gastric cancer and colorectal cancer compared to normal tissues (11–17). In hepatocellular carcinoma, ATG10 levels are markedly higher in Hep3B, HepG2 and PLC cell lines compared to normal LO2 cells (2). Similarly, colorectal cancer cell lines (HCT116, HT29, KM12C, WiDr, LoVo, SW480, SW48, HCT15, DLD1, RKO and CaCo2) exhibit significantly higher ATG10 expression than CCD841 cells (16). Moreover, in cancer tissues and cells, there is a significant trend of increased ATG10 expression levels (**Table 1**).

**Table 1:** ATG10 overexpression in cancer tissues and cells.

Cancer type	Tissues	Size	Cancer cells	Cancer cell lines	Normal cells	Ref
EC	Over	20	–	–	-	(11)
HCC	Over	8	Over	Hep3B, HepG2, PLC	LO2	(12)
ALL	Over	32	–	–	-	(13)
NC	Over	53	–	–	-	(14)
GC	Over	352	–	–	-	(15)
CRC	Over	37	Over	HCT116, HT29, KM12C, WiDr, LoVo, SW480, SW48, HCT15, DLD1, RKO, CaCo2	CCD841	(16)
CRC	Over	20	-	-	-	(17)

EC, Endometrial cancer; HCC, Hepatocellular carcinoma; ALL, Acute leukemia; NC, Nasopharyngeal carcinoma; GC, Gastric cancer; CRC, Colorectal cancer; Over, Overexpression.

## Overexpression of ATG10 acts as an oncogenic factor in the growth of cancer cells

Both *in vitro* and *in vivo* studies indicate that ATG10 functions as an oncogenic factor in cancer progression (**Table 2**). ATG10 overexpression enhances the proliferation of endometrial cancer (HEC-1-A), hepatocellular carcinoma (Hep3B and PLC), nasopharyngeal carcinoma (HONE-1, CNE-2 and 5-8F), colorectal cancer (SW480, SW620 and DLD-1), gastric cancer (SGC-7901, MGC-803, GIST-882, GIST-T1, AGS and HGC27) and lung cancer (A549 and H1299) cells. Furthermore, it inhibits apoptosis in colorectal cancer (SW480, SW620 and DLD-1) cells. *In vivo* experiments confirm that ATG10 overexpression increases tumorigenicity in gastric cancer (SGC-7901) and nasopharyngeal carcinoma (CNE-2) cells in nude mice.

**Table 2:** *In vitro* functional characterization of ATG10 in cancer.

Cancer type	Proliferation	Apoptosis	Migration	Invasion	Cancer cells	Ref
EC	Promotion	–	Promotion	–	HEC-1-A	(11)
HCC	Promotion	–	Promotion	Promotion	Hep3B, PLC	(12)
NC	Promotion	–	Promotion	–	HONE-1	(14)
CRC	Promotion	–	–	–	HCT116	(16)
CRC	Promotion	Inhibition	–	–	SW480	(17)
CRC	Promotion	Inhibition	–	–	SW620, DLD-1	(18)
CRC	–	–	Promotion	Promotion	HCT116, RKO	(19)
GC	Promotion	–	–	–	SGC-7901, iMGC-803	(20)
GC	Promotion	–	–	–	GIST-882, GIST-T1	(21)
GC	Promotion	–	–	–	AGS, HGC27	(22)
NC	Promotion	–	Promotion	Promotion	CNE-2, 5-8F	(23)
LC	Promotion	–	Promotion	–	A549, H1299	(24)

EC, Endometrial cancer; HCC, Hepatocellular carcinoma; ALL, Acute leukemia; NC, Nasopharyngeal carcinoma; GC, Gastric cancer; CRC, Colorectal cancer.

## Overexpression of ATG10 acts as an oncogenic gene in cancer cell metastasis and sensitivity to platinum-based drugs

ATG10 overexpression has been shown to promote cancer metastasis *in vitro* (**Table 2**). Specifically, it enhances the migration and invasion of hepatocellular carcinoma (Hep3B and PLC), colorectal cancer (HCT116 and RKO) and nasopharyngeal carcinoma (CNE-2 and 5-8F) cells (**Table 2**). Additionally, it promotes the migration of endometrial cancer (HEC-1-A), nasopharyngeal carcinoma (HONE-1) and lung cancer (A549 and H1299) cells (**Table 2**). Notably, ATG10 overexpression also induces resistance to oxaliplatin in colorectal cancer (SW480) cells (17).

## The mechanisms by which ATG10 is involved in cancer cell growth and metastasis

**Figure 1:**
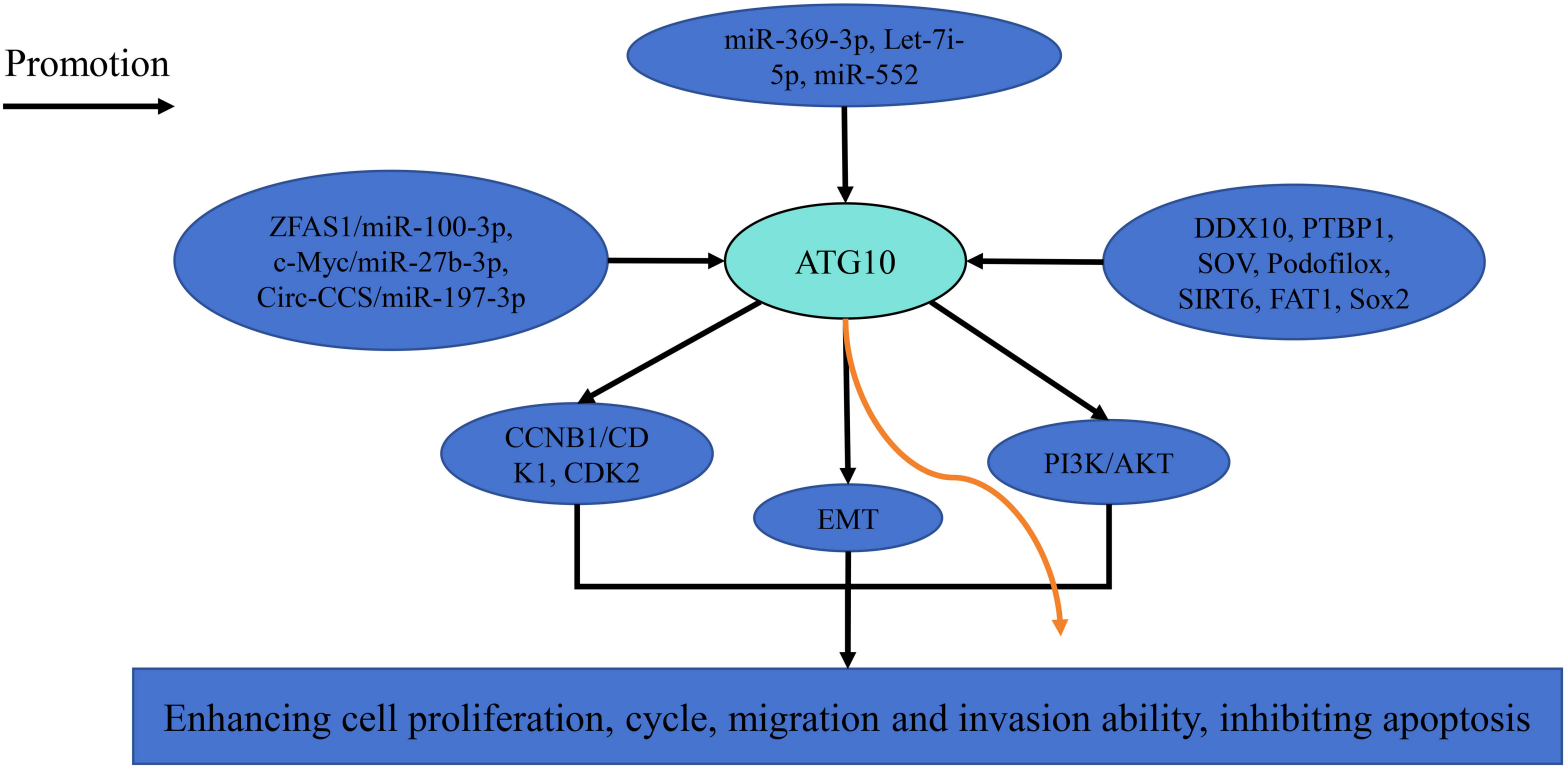
The related pathways of ATG10 in cancer.

ATG10 promotes cancer growth and metastasis through multiple signaling pathways (**Figure 1**). Downregulation of ATG10 suppresses hepatocellular carcinoma cell proliferation, migration and invasion by modulating cyclin B1, CDK1 and CDK2 expression (12). In nasopharyngeal carcinoma and ovarian cancer, ATG10 inhibition disrupts the PI3K/AKT signaling pathway and epithelial-mesenchymal transition, respectively (14, 25). ATG10 expression is regulated by various microRNAs, including miR-369-3p, miR-100-3p, miR-27b-3p, miR-197-3p, let-7i-5p and miR-552 (**Table 3**) and can be suppressed by factors such as DDX10, PTBP1, sodium orthovanadate (SOV), podofilox, SIRT6, FAT1 and SOX2 (18–20, 22, 26–28). For instance, Liu et al. reported that miR-369-3p is downregulated in endometrial cancer, and its overexpression targets ATG10 to inhibit endometrial cancer cell proliferation and migration (11). Similarly, Peng et al. demonstrated that lncRNA ZFAS1 upregulates ATG10 by competitively binding miR-100-3p, thereby promoting nasopharyngeal carcinoma progression via the PI3K/AKT pathway (14).

**Table 3:** ATG10-miRNAs signaling pathways in cancer.

miRNAs	Validated methods	Cancer type	Ref
miR-369-3p	luciferase reporter assay, RT-PCR, WB	EC	(11)
miR-100-3p	luciferase reporter assay, RT-PCR, WB	NC	(14)
miR-27b-3p	luciferase reporter assay, WB	CRC	(17)
miR-197-3p	luciferase reporter assay, WB	GC	(21)
let-7i-5p	luciferase reporter assay, WB	NC	(23)
miR-552	luciferase reporter assay, RT-PCR, WB	Ovarian cancer	(25)

EC, Endometrial cancer; NC, Nasopharyngeal carcinoma; GC, Gastric cancer; CRC, Colorectal cancer.

## Overexpression of ATG10 indicates a poor prognosis for patients with cancer

ATG10 overexpression has been associated with poor prognosis and adverse clinicopathological features in gastric cancer, colorectal cancer and acute leukemia, as determined by RT-PCR, immunohistochemistry and western blotting (**Table 4**). In gastric cancer, elevated ATG10 expression correlates with reduced overall survival, lymph node metastasis and advanced TNM staging (15). Similarly, in colorectal cancer, ATG10 overexpression is linked to poorer overall and disease-specific survival, as well as increased lymphovascular invasion and lymph node metastasis (16). Moreover, in acute leukemia, high ATG10 expression is significantly associated with elevated white blood cell counts (13). These findings underscore ATG10’s potential as a prognostic biomarker and suggest that its inhibition may improve patient survival.

**Table 4:** Overexpression of ATG10 is associated with poor prognosis and clinical features in patients with cancer.

Cancer type	Prognostic indicator	Associated clinical features	Ref
ALL	-	WBC	(13)
GC	OS	Lymph node metastasis, TNM stage	(15)
CRC	OS,DFS	Vascular invasion, Lymph node metastasis	(16)

ALL, Acute leukemia; GC, Gastric cancer; CRC, Colorectal cancer.

## Conclusion

ATG10 is abnormally overexpressed in multiple cancers, including gastric cancer, colorectal cancer and acute leukemia, and its overexpression is strongly associated with poor prognosis and adverse clinicopathological features. Functionally, ATG10 promotes cancer cell proliferation, migration and invasion, underscoring its potential as a tumor biomarker. Mechanistic studies suggest that ATG10 contributes to tumor development by regulating cell cycle-related proteins, epithelial-mesenchymal transition and interactions with miRNAs. As a potential therapeutic target, ATG10 has garnered increasing attention, with ongoing research exploring the effects of its inhibition on cancer treatment. However, current findings are largely based on *in vitro* studies, with limited *in vivo* validation. While preliminary mechanistic insights have been gained, the functional specificity of ATG10 across different cancer types remains unclear. Additionally, clinical studies on ATG10 are scarce, hindering its validation as a reliable biomarker in patient populations. In summary, ATG10 exhibits significant biological functions in cancer progression and holds promise as a novel therapeutic target. However, further systematic *in vivo* studies and clinical investigations are needed to fully elucidate its role in tumorigenesis and its potential in targeted therapy.
